# Methicillin Resistant Staphylococcus Aureus Infection as a causative agent of fistula formation following total laryngectomy for advanced head & neck cancer

**DOI:** 10.1186/1758-3284-2-14

**Published:** 2010-06-28

**Authors:** Jean-Pierre Jeannon, Ahmad Orabi, Argyris Manganaris, Ricard Simo

**Affiliations:** 1 Department of Ear Nose & Throat - Head and Neck Surgery, Guy's and St Thomas' Hospital NHS Foundation Trust, London, UK

## Abstract

**Aims:**

The purpose of this paper was to investigate the impact of Methicillin Resistant Staphylococcus Aureus (MRSA) infection in the aetiology of pharyngo-cutaneous fistula (PCF) formation following total laryngectomy for advanced laryngeal cancer.

**Methods:**

This was a retrospective uncontrolled case study series of 31 consecutive patients based in a single institution tertiary referral head and neck oncology centre.

**Results:**

Pharyngo-cutaneous fistulas (PCF) following total laryngectomy occurred in 10 (32%) patients. MRSA was identified in 80% of patients with a PCF compared to 9% of patients that did not develop a fistula (*p *= 0.0001255 Fisher exact test). MRSA infection (*p *= 0.00012) and previous radiotherapy (*p *= 0.00025) were the only significant factors found to be important in fistula formation on multivariate analysis. Post-operative infections such as cellulitis, chest infection and carotid fistula were also associated with MRSA infections.

**Conclusion:**

MRSA infection following total laryngectomy for laryngeal cancer can lead to potential serious complications such as PCF. Patients who underwent total laryngectomy following radiotherapy failure are at a higher risk of acquiring MRSA.

## Introduction

Total laryngectomy is a major surgical procedure used to treat advanced cancer of the larynx.

**Figure 1:**
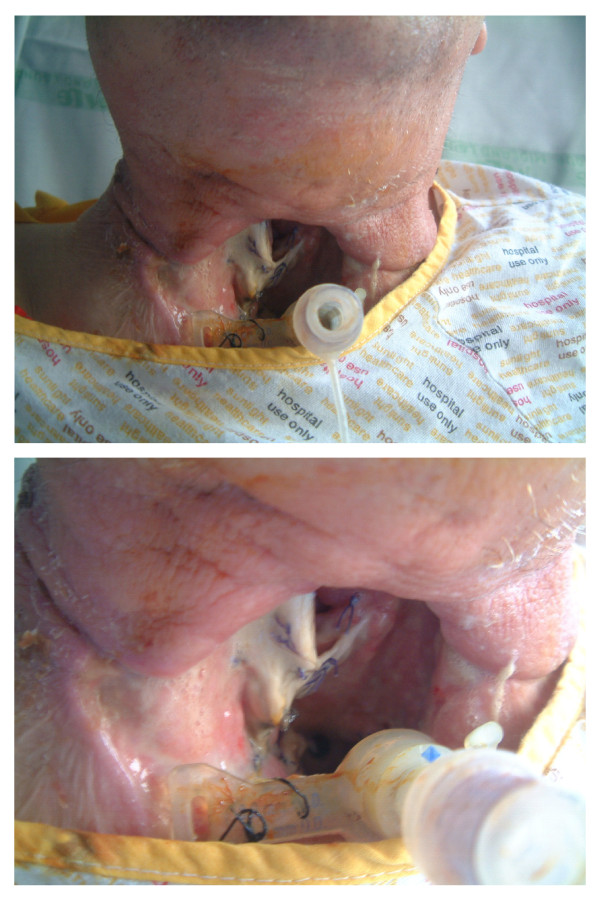
**Photograph showing large PCF 10 days post SL with heavy growth of MRSA**.

Pharyngo-cutaneous fistula (PCF) is a complex type of wound dehiscence and hence a potential major complication following total laryngectomy. The reported incidence of this complication following laryngectomy varies between 8-40% [1]. It results when a pathological communication develops between the internal mucosa of the pharynx and the external skin which discharges saliva (Figure 1). The immediate sequelae of PCF is that oral feeding has to be suspended. This delays healing in the post-operative period and the subsequent loss of fluids, electrolytes and proteins in the fistula further compromises the patient's condition. Long-term problems following PCF may include aspiration, further wound infection, chest infection and cachexia.

The aetiology of PCF is thought to be multi-factorial [1]. Several factors have been thought to be important and can be generally divided into patient factors and operative factors. In particular these can include pre-operative co-morbidity, pre-operative radiotherapy, peri-operative wound infection, advanced T stage and closure technique.

Methicillin Resistant Staphylococcus Aureus (MRSA) infection in the post-surgical patient is associated with higher post-operative morbidity including skin breakdown and cellulitis [2]. No previous study has investigated MRSA infection as a causative factor in PCF following laryngectomy.

The objective of this paper is to review our experience of patients who have developed PCF following total laryngectomy and determine the influence of MRSA is a causative factor.

### 'What is already known about this topic?

The aetiology of pharyngo-cutaneous fistula (PCF) is thought to be multifactorial. MRSA infection is associated with higher postoperative morbidity including skin breakdown and fistula formation. No previous study has investigated MRSA infection as a causative factor in PCF following laryngectomy.

### 'What does this article add?

This is the first study to identify MRSA as a possible causative agent in PCF following laryngectomy. Implementation of MRSA eradication protocol is recommended in every patient undergoing laryngectomy.

## Material and Methods

This is a retrospective review of 31 consecutive patients who underwent total laryngectomy at Guy's & St Thomas NHS Foundation Trust London, which is the tertiary regional cancer centre for the South East London Head and Neck Cancer Network (SELCN). The study period ran from January 2004 until January 2007. From January 2004 all patients undergoing treatment for head and neck cancer have been entered prospectively on a database.

All patients with a diagnosis of head and neck cancer were discussed in the regional head and neck oncology multidisciplinary team (MDT) meeting prior to definitive treatment being commenced.

Patients underwent elective pre-admission MRSA screening upon admission through swab taking. Subsequent screening continued throughout the in patient stay in hospital. Therefore all new MRSA acquisitions were therefore detected prospectively. During their inpatient stay as a routine protocol, patients were screened for MRSA twice weekly.

Patient data including MRSA swab results and post-operative water soluble swallow tests were retrieved from the electronic patient record.

All patients undergoing head and neck surgery were given peri-operative antibiotic prophylaxis and post-operative gastro-oesophageal reflux medication as a protocol.

For the purpose of this study a pharyngo-cutaneous fistula was defined as a clinically detected fistula or radiologically identified leak that delayed the onset of oral feeding after laryngectomy.

Multivariate analysis was performed using STATA statistical software to analyse the data. The following factors were analysed to determine their effect on fistula formation: T stage, previous radiotherapy and MRSA infection.

### Ethical Considerations

Data was retrieved from the database using medical record numbers hence patients remained anonymous. Ethical approval was not needed for this study.

## Results

During the study period (2004 to 2007 inclusive), 31 total laryngectomies were performed for advanced laryngeal cancer.

The patient demographic data were illustrated in Table 1. The mean patient age was 65 years (range 37 to 86).

**Table 1:** Patient Demographic Data

Patient	TNM	PREVIOUS RADIOTHERAPY	MRSA	FISTULA
**1**	T4N0M0	N	X	X
**2**	T2N0M0	Y	√	√
**3**	T4N1M0	N	X	X
**4**	T2N0M0	Y	√	X
**5**	T4N0M0	Y	X	X
**6**	T3N2BM0	N	X	X
**7**	T4N1M0	N	X	X
**8**	T3N2BM0	N	X	X
**9**	T4N0M0	N	√	√
**10**	T4N0M0	N	√	√
**11**	T3N1M0	N	X	X
**12**	T4N2CM0	N	X	X
**13**	T3N2CM0	Y	√	√
**14**	T4N0M0	N	X	X
**15**	T3N0M0	Y	√	√
**16**	T2N0M0	Y	X	√
**17**	T2N0M0	Y	X	X
**18**	T4N0M0	N	X	X
**19**	T3N0M0	Y	√	√
**20**	T4N1M0	Y	√	√
**21**	T2N0M0	Y	X	X
**22**	T4N0M0	Y	X	√
**23**	T4N2BM0	N	X	X
**24**	T3N0M0	Y	X	X
**25**	T3N1M0	N	X	X
**26**	T2N0M0	Y	X	X
**27**	T2N2BM0	N	√	√
**28**	T2N0M0	Y	X	X
**29**	T2N0M0	Y	X	X
**30**	T3N2BM0	N	√	X
**31**	T2N0M0	Y	X	X

15 patients underwent primary laryngectomy for advanced laryngeal cancer and 16 patients had salvage laryngectomy for recurrence after previous failed radiotherapy.

A pharyngo-cutaneous fistula (PCF) occurred in 10 (32%) patients, MRSA was identified in 8 of these patients. Of the 21 patients that did not develop a PCF, 2 were found to acquire MRSA infection (in the tracheal stoma). This difference reached statistical significance *p *= 0.0001255 Fisher exact test (Table 2).

**Table 2:** MRSA Infection and Fistula Formation

	FISTULA +VE	NO FISTULA	TOTAL
**MRSA +VE**	8	2	10

**MRSA -VE**	2	19	21

**TOTAL**	10	21	31

(*p *= 0.0001255 Fisher exact test)

None of these 31 patients were found to be carriers of MRSA on pre-operative screening. Therefore from this group of 31 patients, 10 (32%) were seen to acquire MRSA during their hospital stay.

Using multivariate analysis, previous radiotherapy (*p *= 0.00025) and MRSA infection (*p *= 0.00012) were identified as significant risk factors for PCF (Table 3).

**Table 3:** Multivariate analysis of Risk Factors for PCF

	*p *VALUE	ODDS RATIO	CONFIDENCE INTERVAL
T STAGE	*p *= 0.054	1.15	0.4 - 4.5
MRSA INFECTION	*p *= 0.00012	9.2	2.36-35.8
PREVIOUS RADIOTHERAPY	*p *= 0.00025	8.6	1.24-27.9

(Log rank test)

**Figure 2:**
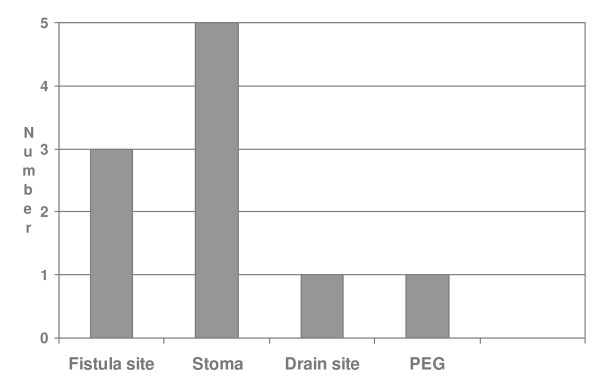
**Sites where MRSA was isolated**.

MRSA was cultured from the tracheostomy stoma site in 5 patients, in 3 patients it was detected in the fistula site, from the surgical drain 1 and from the gastrostomy site in 1 patient (Figure 2).

The 2 patients that did acquire MRSA but did not develop PCF did however suffer significant morbidity. 1 patient developed a significant cellulitis infection and 1 had a carotid fistula that required return to the operating theatre to arrest the bleeding.

## Discussion

MRSA may reside in healthy individuals and not cause any health problems. However hospital acquired infection can result in significant morbidity and mortality. The first case of MRSA was identified as early as 1961- 1962 shortly after introduction of Methicillin in 1960 [2-4]. MRSA has caused particular concern due to the resistance to standard antibiotics caused by gene mecA and the rapidity with which it spreads [2-5]. MRSA arose as a hospital based infection but has steadily spread to the community [4,5]. Hospital acquired MRSA infection has been steadily increasing and in some reports infection rates have reached over 60% [4]. In this series we have identified an acquisition rate of 30% which is high. We believe this is because head and neck patients undergoing major surgery particularly at risk of this infection.

Head and neck cancer patients are prone to MRSA infection due to a number of factors such as: prolonged hospitalization, intravascular catheterization, compromised host immunity, malignancy, chemotherapy, radiotherapy surgery, prior antibiotic therapy and prolonged operative time [2-7]. Many of these factors were present in our series of patients. Following total laryngectomy, the mucosa of the trachea is permanently directly exposed to room air and we feel this site is particularly prone to MRSA infection. This is why the tracheal stoma was shown to be the commonest site of infection.

Serious postoperative complications related to MRSA infection have been identified in head and neck surgery patients with significant increase in morbidity [8,9] number of surgical procedures and prolonged hospitalization time [10]. One of our patients had a carotid fistula or 'blow out' which is a life threatening complication. Colonisation of the tracheostomy site appeared to be the most important site of MRSA infection. The creation of a permanent tracheostomy following total laryngectomy may significantly reduce the patient's local defence mechanism allowing a conduit of infection to the aero-digestive tract.

PCF following laryngectomy constitutes a particularly serious complication with subsequent devastating medical, functional, psychological and economical effects. The rate ranges considerably from 8% to 40%, according to the literature [11-17]. A recently published meta-analysis of previously published studies on post-laryngectomy PCF concluded that the following four factors were thought to be significant: a) preoperative radiotherapy, b) postoperative haemoglobin level below 12.5 g/dl, c) prior tracheostomy, and d) preoperative radiotherapy and concurrent neck dissection [1]. Our study also has identified pre-operative radiotherapy as an important risk factor for PCF.

To our knowledge this is the first study to investigate and suggest a potential causal relationship between PCF and MRSA in patients undergoing total laryngectomy for laryngeal cancer. Whilst we have found a significantly higher MRSA infection rate in patients that have developed PCF following laryngectomy compared to those that did not, this association only implicates MRSA as a possible causative factor in. We recognise the limitations of this paper in that the design is retrospective and sample size small, however we feel the high incidence of MRSA infection seen in the PCF patients warrants further study.

The incidence of PCF in our laryngectomy patients was high (32%) compared to previously published series. The focus of future study in our institution is aimed at reducing this PCF rate.

MRSA principal mode of transmission is through direct contact via hospital personnel and by airborne transmission particularly from patients with tracheostomies [2-6]. Many strategies have been advocated to prevent MRSA infection with variable degree of evidence based, including search and destroy policy, restrictive antibiotic prescribing policy [12], hand hygiene with the use of alcohol- based solutions, isolation measures [13]. The high incidence of MRSA infection seen in this patient group obliged us to upgrade our infection control protocols and we have subsequently seen a dramatic reduction in MRSA infection rates.

There is some controversy regarding treating MRSA colonization versus infection particularly in hospital staff carriers and in endemic areas like UK, which can be proved to be difficult. However because of the particular risks associated with head and neck cancer patients, colonized patients should be eradicated prior to major surgery [10]. The current approach is to carry out preoperative screening and appropriate treatment by antiseptic skin washes, nasal mupirocin and chlorhexadine mouthwashes, isolation or barrier nursing and perioperative anti MRSA antibiotic in carrier patients, and judicious antibiotic prescription practice.

Having carried out this study, we have implemented an MRSA eradication protocol in all patients undergoing laryngectomy even if they are not colonized with MRSA in order to reduce infection rates. We have also seen a significant overall reduction in MRSA infection rates across the hospital due to strict hand hygiene, screening and MRSA eradication protocols being implemented.

MRSA will continue to pose a challenge because of rising incidence not only in hospitals and nursing homes but also in the outpatient community. The emergence of increasingly resistant staph. aureus organisms and the demand to treat patients with more and more complex medical conditions will continue to test medical and nursing staff in the future.

## Conclusion

This is the first study to identify MRSA as a possible causative agent in pharyngo-cutaneous fistula (PCF) following total laryngectomy for laryngeal cancer. Patients who underwent laryngectomy following radiotherapy failure and acquired MRSA were at higher risk of PCF. MRSA infection post laryngectomy can lead to significant morbidity. A multidisciplinary approach is essential for effective management. Implementation of MRSA eradication protocol is recommended in every patient undergoing salvage laryngectomy.

## Conflict of interest

The authors declare that they have no competing interests.

## Authors' contributions

JPJ conceived the study and wrote the manuscript, AO and AM participated in the design of the study and performed the statistical analysis and RS revised the manuscript. All authors read and approved the final manuscript.
